# Development and Evaluation of an Artificial Intelligence–Based Cognitive Exercise Game: A Pilot Study

**DOI:** 10.1155/2022/4403976

**Published:** 2022-09-27

**Authors:** Sung-Jong Eun, Eun Joung Kim, Jung Yoon Kim

**Affiliations:** ^1^Digital Health Industry Team, National IT Industry Promotion Agency, Jincheon, Republic of Korea; ^2^Culture Contents Technology Institute, Gachon University, Seongnam-si 13120, Gyonggi-do, Republic of Korea; ^3^Department of Game Media, College of Future Industry, Gachon University, Seongnam-si 13120, Gyeonggi-do, Republic of Korea

## Abstract

Recently, cognitive serious games have successfully been employed to train cognitive abilities in elderly people with mild cognitive impairment, Alzheimer's disease, and related disorders. However, despite the continuous rehabilitation game design and its applications, the existing cognitive exercise games fall short of user interaction and personalized elements with regard to difficult levels, which leads to users leaving early and losing interests during the gameplay. In this regard, the purpose of the study was to design and develop the serious game inclusive of playful elements for user motivation, the web-based mobile application system for easy accessibility, and Artificial Intelligence– (AI–) based difficulty level adjustment system for prevention from earlier leaving out in the middle of the play so that the elderly users can feel entertaining and immersed into the cognitive game voluntarily. This study was designed as an eight-week pilot experiment with thirty-seven participants in their 60s to 80s for the game's usability assessment purpose. Results of the study showed that the AI-based cognitive exercise game was acceptable, interesting, and motivating for the elderly people and the test results before and after the eight-week training suggest a relationship between longer the training on the game and lower cognitive assessment scores including geriatric quality of life scale, geriatric depression scale, and Korean version of mini-mental state examination (MMSE). These correlations demonstrate the potential value of serious games in clinical assessment of cognitive status for the elderly users with varying cognitive ability. Based on these results, the elderly-centered serious game with playful element can be potentially used in clinical settings, allowing the cognitive training to be more enjoyable and more medically effective. Given these promising results, a more focused study can extend to the game system or additional game tools or features to be explored that solely target the elderly by applying AI and advanced visualization devices.

## 1. Introduction

As global life expectancy at birth has been rising with remarkable speed, aging-associated diseases are also sharply increasing. Faced with fierce competition, people today experience ongoing stress and anxiety which excessively stimulates and strains brain activities leading to cognitive malfunctions such as early signs of dementia [1–4]. Different from other diseases, degenerative brain diseases such as Alzheimer's among age-related cognitive disorders are destructive to the quality of life, and the patient and caregivers carry a high financial, social, and emotional burden due to those chronic harmful effect [5]. However, it is difficult to reveal causes of neurodegenerative disorders mainly because of the complexity of the brain, and thus, few clear medical treatments have been practiced despite much research having been done for decades [6].

Recent medical research suggests that elderly people do brain training or cognitive therapy exercises that require user interactions, which are effective for the enhancement of cognitive functions [7, 8]. Cognitively-engaging exercises with various modality and intensity of physical exercise appear to have a strong effect on older people with mild cognitive impairment [9]. Accordingly, healthcare digital serious games have been designed to engage and train brain regions that are responsible for everyday functioning including executive functions, domains of memory, and processing. The experimental studies on serious games specifically adapted to people with neurodegenerative disorders show that physical games can positively affect the players with mild Alzheimer's disease and mild cognitive impairment [10, 11] such as attention and memory [12] and visual-spatial abilities [13]. In addition, these serious games can have a positive impact on mental health, for instance, to prevent or treat anxiety and depression [14, 15]. These initial results seem promising yet variations exist in the effectiveness of the cognitive serious games because the existing cognitive training falls short of the focuses on its targeting users and individual customization. Most of all, the older adults have difficulties in being familiar with the game tools and complex user interface or the game requires too high skills or demanding performances [16]. Thus, there is a much need for a user-centered exercise game, namely, the serious game that targets specifically the elderly people so that the elderly users can keep on playing the healthcare game with higher encouragement until they can see the clinical effects. In line with this, the study was conducted to design an Artificial Intelligence– (AI–) based cognitive training game that can automatically adjust the difficulty level according to the individual's performance to enhance their voluntarily engagement and eventually to impact on the clinical effects. The study did an empirical research as a pilot experiment with thirty-seven participants in their 60s to 80s during eight-week training for assessment purpose.

## 2. Literature Review

A serious game is designed for a primary purpose other than just pure entertainment. It excludes the negative aspects of game such as gambling, violence, and addiction but focuses on its positive sides including immersion, fun, and educational effectiveness to attain its primary purpose. Serious games are widely being used for education, industrial simulation, marketing, emergency management, and healthcare as shown in Table 1.

Among those applied fields, healthcare serious games take an important role. Ben Sawyer, one of the leading researchers in serious games, predicted that healthcare games will be exponentially growing and widely applicable across healthcare simulation education and training [17]. The following are the recent successful examples of healthcare serious games. Hungry Red Planet (http://www.hungryredplanet.com) attempts to make learning about nutrition fun for kids by sliding the educational component into a sci-fi and turn-based strategy game with an interesting storyline. It is an educational game that focuses on the importance of nutrition and healthy eating and, further, can help cure nutrition-associated diseases [18]. Dance Dance Revolution [19] is an indoor exercise video game that uses dance platforms or bicycles, while the FreeDive developed by BreakAway Games (http://www.breakawaygames.com) aims to effectively distract children when they undergo frequent and often painful medical procedures as well as bring joy to chronically ill children [20]. S.M.A.R.T BrainGames (http://www.braingames.com) and Full Spectrum Warrior (http://www.fullspectrumwarrior.com) are intended for patients with mental illness or post-traumatic stress disorders allowing them to do cognitive training and interactive brain exercises, thus strengthening their mental skills [21]. These rehabilitation serious games can improve motor activities such as walking, sitting, standing, and the process of switching from one type to another for post-stroke patients [22].

In South Korea, there are some representative cognitive exercise games as shown in Table 2. The “Village that is getting into younger” is a brain training game wherein its medical effectiveness was confirmed with people that are aged over 65 years for six months at Asan Medical Center in Seoul and is being used in twenty-five welfare facilities. On the other hand, the “Paldogangsan” developed by Hoseo University is an exercise game in which users can sit, walk, and exercise while watching the beautiful national and provincial scenic areas. It features the elements of health training such as memory performance and physical enhancement.

The existing cognitive game systems were successfully employed to train physical and cognitive abilities for the elderly users compared to the exercise games in the market including the Nintendo Wii Fit and Microsoft Your Shape that are considered too demanding and can be risky for elderly people since they mainly target on the healthy users [23]. However, despite the continuous healthcare game design and its applications, the existing cognitive exercise games fall short of user interaction and personalized elements with regard to difficult levels, which leads to users leaving early and losing interests during the gameplay. It is noted that the game system that keeps the users playing the exercise games is a very important factor to see meaningful medical results [24]. Thus, when designing a serious game for cognitive training, combining playful elements and appropriate medical simulation is necessary for the users to continue the gameplay. In this regard, the purpose of the study was to design and develop the serious game with an AI-based personalized system that adjusts the difficulty level of each cognitive exercise drill according to the user's performance so that the elderly users can feel entertaining and immersed into the cognitive game voluntarily.

In the review of the empirical studies that report the serious games' positive impact on the cognitive abilities, they keep track of participants' performance overtime and use the Mini-Mental State Examination (MMSE) and other cognitive assessment methods with a pilot study [24]. And most of studies focus on the patients suffering from Alzheimer's disease and other related disorders [25], but there remains a need for a broad range of older adults with varying cognitive ability. As a start of collecting incremental data, this study was carried out on a relatively small pilot sample of thirty-seven people to test and evolve usability and usefulness of the serious games, and the participants were the healthy individuals in their 60s and 80s to test the game that aims to prevent cognitive diseases and maintain their well-being.

## 3. Development of a Healthcare Serious Game with Cognitive Exercise Drills

### 3.1. Four Domains of Cognitive Functions

The four basic cognitive functions include judgment, memory, quick adaptation, and concentration. Considering these basic cognitive functions, the elderly people's cognitive areas were divided into four domains of attention, logic, response time, and memory and defined each part including cognitive target abilities as shown in Table 3 [26].

To be particular, attention includes cognitive abilities such as “two-dimensional mental rotation calculation” and “attention and concentration.” Logic includes abilities related to “logical solution” and “identifying space-time location”. Response time includes “response behavior” and “boundary judgment,” and memory includes “impression memory” and “status memory.”

### 3.2. Design Six Cognitive Exercise Drills

Three steps were conducted to design the proposed cognitive exercise game: (1) designing the cognitive drills by including the playful elements, (2) developing the web-based game system for user accessibility, and (3) applying an AI-based data analysis technology to make a difficulty level adjustment. As mentioned earlier, four cognitive functions were categorized that include attention, logic, response time, and memory, and each function-related drill was developed to improve hand movements, memory, concentration, vision-based identification, recognition, and graphic interpretation. For a better customized design and medical effectiveness, the healthcare digital game was made inclusive of playful elements, for user motivation, a web-based mobile application system for easy accessibility, and personalized elements for prevention from leaving out in the middle of the play.

### 3.3. Designing the Cognitive Drills by including the Playful Elements

The digital cognitive game is displayed to the target players (i.e., elderly people in their 60s to 80s), so the user-centered game contents goes into the fundamentals of game design. In order for the elderly to get encouraged and keep motivated, the entire game consists of six simple mini games in which they are able to interact with moving objects, different types of symbols, and numbers.

In the game, users initially provide their personal information (including their gender, age, and weight) to create an ID account, and they perform the six different drills in the form of mini games as shown in Table 4. The mini games are displayed to the user according to their difficulty level and performance complexity.

Consultation from psychiatrists about the elderly people's IT device control abilities were also received when designing the entire game structure, drills, and UI/UX to be easily accessible and operative.  Table 5 illustrates the flow of the six drills to be displayed and their detailed explanations.

### 3.4. Developing the Web-Based Game System for User Accessibility

The proposed cognitive training game was designed and developed on a web-based system. The web-based system is easier for users to access, and it is also informative for the developer to manage the database of individual users and their history of gameplay performance. The healthcare game system was designed by using a touch screen where the player's hand movements can be identified and measured, which is different from other cognitive games with a mouse click and simple finger touch. It assumes that human cognitive, physical, and emotional functions are not separately performed but closely correlated so that a motion-based game is more effective than the existing cognition-based games for the purpose of the elderly's degenerative brain malfunction.

**Figure 1:**
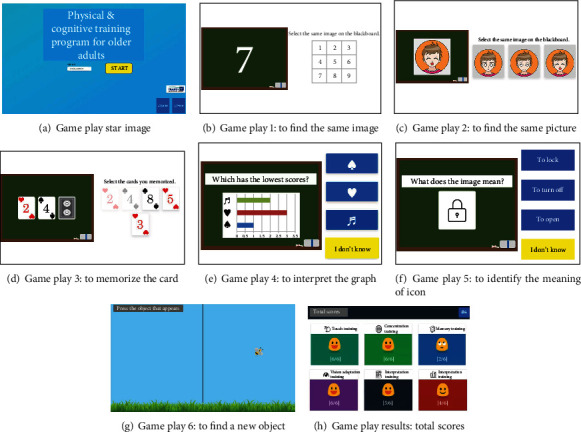
The final version of the designed cognitive drills.

The game system can run with the HTML5 browser with the Web content standard, and it is applicable for PC, smartphones, tablets, and other handheld devices. Its development consists of three steps including DB development, back-end development, and client development as shown in Table 6. The game's data model is NoSQL document-based databases [27], it uses Firebase to build the back-end [28], and the Angular JS framework is used as the front-end development tool to optimize the transitional speed between the applied programs [29]. The game uses Bootstrap design to provide an appropriate layout to the users' device [30]. The final version of each drill is shown in Figure 1.

### 3.5. Applying AI-Based Data Analysis to Make a Difficulty Level Adjustment

The proposed cognitive exercise game offers an adjustment system according to the individual user's performance of the gameplay, which is effective both in keeping them focused on the game during a certain period of time and eventually making meaningful data results to validate its cognitive effectiveness. Initially, the basic structure of the adjustment system was designed based on the collected data from the users in the pretest. The system has two procedures: first, to decide whether the user needs a difficulty adjustment or not and second, if so, whether to adjust its level higher or lower.

When deciding whether to adjust or not the difficulty level, a long short-term memory– (LSTM–) based recurrent neural network (RNN) architecture was used [31]. The dataset consists of a total of fifty users' data for testing, which were divided into two groups. One group of data needed level adjustment, and the other group needed no level adjustment. The first level-adjustment group data was half for level-up and the other half for level-down. Data input included the problem-solving time, total scores, and scores for each question. The module with the least error value was selected for machine-learning to deduce an optimal model. Thus, the AI adjustment system decided whether to stay at the same level, go to a higher level, or to the lower level.

**Figure 2:**
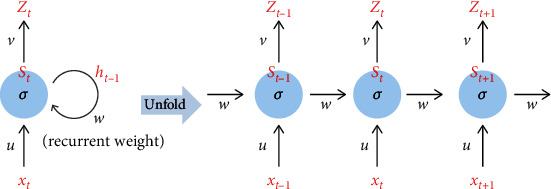
The basic RNN architecture.

RNN is a deep learning algorithm that is effective in recognizing the sequential characteristics of data using patterns through repetitive structure with sequential data or time series data.  Figure 2 shows the basic architecture in which the hidden layer goes to itself, which is named recurrent weight (W). It is the structure to recognize the repetitive patterns from the dataset and to analyze the current status by finding the optimal value of W.

**Figure 3:**
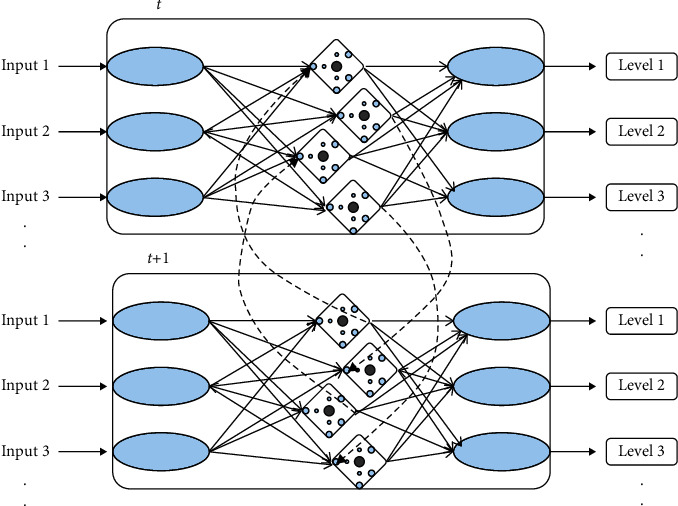
The entire network architecture according to time.

As shown in Figure 3, the designed RNN architecture decides whether to adjust the difficulty level based on the calculation of node connectivity between the current point (t) and the next point (t +1), which is the way to analyze the user's past performance.

After deciding whether to make an adjustment, the system decides which elements should be adjusted in each mini game. As shown in Table 7, each mini games or drills have different items to be adjusted with one level for each time.

## 4. Empirical Assessment of the AI-Based Cognitive Exercise Game

An empirical study was conducted through a tablet device for eight weeks targeting thirty-seven people in their 60s to 80s in order to assess the effectiveness of the proposed AI-based cognitive exercises. This prospective observational clinical study with participants recruited from the Michuhol Senior Welfare Center located in Incheon Province, South Korea, under a research protocol approved by the Gachon Hospital. Clinical research assistants (RAs) administered the interview to recruit the appropriate participants with the exclusion criteria that included patients who were critically ill, receiving psychoactive medications, blind, or unable to speak and communication verbally. The survey details are shown in Table 8.

A total 39 participants (5 males and 34 females) were recruited between the 60s and 80s. Two participants were excluded for not playing the serious game and not completing any of the cognitive assessments. The participant's demographic information is shown in Table 9. Each participant was instructed on how to play the serious game and interact with the tablet. There was no limit on the number of attempts to play the game, and participants were invited to provide open feedback at the end of the study. The empirical assessment has two parts: the subject's before/after cognitive abilities and their satisfaction and performance related to the cognitive exercise game. After comparing these survey data, a valid conclusion was made and verified the game's effectiveness in a systematic way.

### 4.1. Pre- and Post-Survey

The survey was conducted two times before and after playing the cognitive exercise game with questionnaires and empirical tests to see how effective the eight-week cognitive training was. The types of questionnaires consisted of a basic questionnaire and the participants' perceived health condition, geriatric quality of life scale (WHOQOL) test [32–34], geriatric depression scale (GDSSF-K) [35], a Korean version of MMSE for dementia screening [36–38] and a Korean dementia screening questionnaire-cognition test. In addition, another survey was conducted to keep track of the individual's game performance and evaluate their general satisfaction towards the gameplay.

### 4.2. Quality of Life Test

In the quality of life test, the overall health condition had 2.19 points in the pre-survey and 2.41 points in the post-survey. The individual quality of life was recorded at 2.62 points for pre-survey and 2.86 points for post-survey. The results showed that both overall health condition and overall quality of life tended to increase. Of course, the cognitive gameplay cannot be said to have on positive impact on every item in the players' quality of life test, but as shown in Table 10, one of the reasons for the total increase resulted from “memory and concentration” with the plus 0.22 difference.

### 4.3. Geriatric Depression (GDSSF-K) Test

As shown in Table 11, two questions showed a big difference between the pre- and post-surveys. First, in the question of “Do you often feel that your life is boring?”, more respondents answered “no” from 74.4% to 91.9%. The second question was “Do you feel your memory is inferior to that of your peers?”, more respondents answered “no” from 74.4% to 81.1%.

### 4.4. Korean Version of Mini Mental Status Examination for Dementia Screening

Korean version MMSE dementia screening scores increased from 4.81 points to 5.05 points after playing the cognitive exercises as shown in Table 12.

After playing the cognitive exercises for eight weeks, positive changes were found in the two questions associated with cognitive abilities as shown in Table 13. More respondents have answered “No” with the two questions that include “I often go for something but forget to bring it” and “My calculation abilities have declined.”

### 4.5. Empirical Assessment of the AI-Based Cognitive Exercise Game

In order to assess the effectiveness of the digital cognitive exercises game for the elderly people, a survey of satisfaction and each drill performance was conducted.

### 4.6. Result of Satisfaction Survey

The satisfaction survey consisted of several questions related to fun elements, difficulty level, playtime, and the subjective assessment of cognitive enhancement. The results showed that the average points of all questions went over 4.0 points in the 5-point Likert scale as shown in Table 14. To be specific, in the question of “do you think the game is helpful to advance your cognitive function?”, the point was the highest with 4.62. And for the questions related to gameplay itself, the respondents gave the more than 4.5 points with fun play, easy to play, and adequate playtime. The general difficulty level of the game was 3.19 points, which also showed that the game was difficult enough for aged users to play. Thus, the findings validated the fact that the users were generally satisfied with the cognitive exercise game's playful contents and difficulty adjustment system.

Respondents answered the things to be improved with regard to the game including “how to use a computer,” “more detailed description,” and “image diversification.” These improvements were associated with technological literacy that can be different from individual ability to use, manage, understand, and assess computer skills or game UI. And they were insignificant since only one person for each answered yet the majority of users said that “there is nothing particular” as shown in Table 15. This result indicated that the user satisfaction was very high.

### 4.7. Individual Performance of the Cognitive Exercises Game

As shown in Table 16, the total average of touch training was 5.94 points, concentration training was 5.09 points, memory training was 4.77points, vision adaptation training was 5.65 points, icon training was 4.05 points, and graph training was 5.30 points.

All the training showed better performance except for touch training. For concentration training, the individual performance increased by 0.005 as playtime increased, which seemed quite insignificant but for the memory training, the individual performance got better by 0.026, which was a statistically significant increase. For vision adaptation training, as they played more and more, the scores rose by 0.034, icon training by 0.113, and graph training by 0.042. The reason for the decrease in touch training is due to the too small-sized tablets for the users to play the game. More detailed information for the abovementioned significant four training effect estimation are described in Tables 17–20.

## 5. Result and Discussion

The results of the empirical study confirmed that the AI-based cognitive exercise game was acceptable and satisfactory for the elderly people. This interpretation was confirmed by the fact that thirty seven participants successfully completed the eight week training with only two drop-outs, and by the fact that participants rated the game experience as interesting reported to be highly satisfied and motivated by the game. Thus, the results of the user's general satisfaction suggested that both difficulty level adjustment AI system and the web-based game system for user accessibility played an important role in enhancing the elderly user's motivation and voluntarily participation in doing the cognitive exercises.

The individual's general exercise performance on the serious game became higher as the playtime increased. And the test results before and after the eight week training suggested a relationship between longer the training on the game and lower cognitive assessment scores includes WHOQOL, GDSSF-K, and Korean version of MMSE. These correlations demonstrated the potential value of serious games in clinical assessment of cognitive status for the elderly users with varying cognitive ability. Namely, the AI-based and user-centered cognitive exercises positively impacted the elderly's cognitive abilities in general. The results are in the similar line with other studies that a user-centered design is the key in ensuring that the serious game was usable with a challenging group of elderly patients [24], but the present study implied that the automatic system that is based on AI is a useful technology to incorporate a playful element and directly affect the cognitive enhancement.

Another interesting finding was that the touch training is highly related to the device where the participants play the serious game. Even though these technologies also provide the ability to modify contrast and brightness and text size and font to increase readability but when the screen is small, it affected on the kinetic exercise in a negative way. Some elderly users may be uncomfortable using technology or the device interface is able to hamper their adequate motivation and realistic assessment of ability.

## 6. Conclusion

This study designed the cognitive exercise game inclusive of playful elements for user motivation, the web-based mobile application system for easy accessibility, and AI-based difficulty level adjustment system for prevention from earlier leaving out in the middle of the play. The empirical results suggest that a cognitive serious game incorporated with a user-centered and customized difficulty level system was acceptable, interesting, and motivating for the elderly people and the AI-based serious game that specifically targets the elderly people positively impacted their cognitive abilities in general. Based on these results, the elderly-centered serious game with playful element can be potentially used in clinical settings, allowing the cognitive training to be more enjoyable and more medically effective. Given these promising results, a more focused study can extend to the game system or additional game tools or features to be explored that solely target the elderly by applying Artificial Intelligence and advanced visualization devices. To optimize the advantages of healthcare serious games as cognitive rehabilitation tools, it needs to apply the state-of-art immersive technologies and machine learning algorithms with which the game will be well-designed to deal with distinctive characteristics of the broad range of cognitive disorder patients from initial stage of cognitive decline towards more serious cognitive deteriorations, notably dementia. This further study will provide an innovative medical solution to manage the cognitive health and further to cure the aging-associated brain disorder.

## Figures and Tables

**Table 1:** Serious game applications.

Application	Purpose
Education	Aims for knowledge learning, language acquisition, and behavior training
Simulation	Aims to simulate real-world scenarios for the prevention of accidents or precautions against them
Advertising	Aims to create a demand for products or public awareness of the government policies
Leisure and sports	Aims to enjoy leisure and sports
Healthcare	Aims to enhance and manage physical and mental health

**Table 2:** Healthcare serious games in South Korea.

Organization(product)	Purpose	How to play	
National Rehabilitation Center (kinect-based rehabilitation game)	Exercise therapy for musculoskeletal rehabilitation	Move the points displayed on the screen by hand, and the sounds from the musical instruments are produced	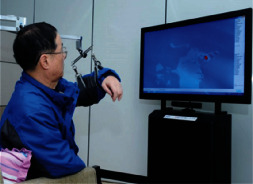
Uniana (village that is getting into younger)	Cognitive training with visual and auditory signals	Use a button or touch screen by hand to move their fingers or hands	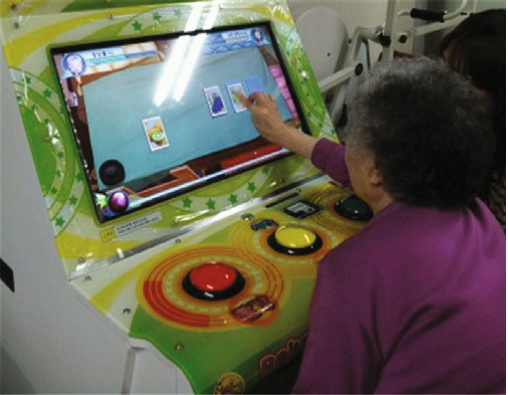
Hoseo University (Paldogangsan)	Memory performance and physical training	Walk, sit, and exercise while looking at the screen with various sceneries	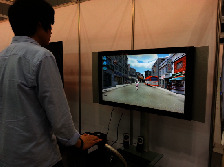

**Table 3:** Four cognitive abilities and their definition.

Domain	Definition	Target abilities	Brain areas
Attention	Ability to selectively concentrate on a discrete aspect of information and deal with it	Divided attention, attention, concentration, spatiotemporal processing	Posterior lobus parietalis, frontal lobe
Logic	Ability to think and decide in accordance with reasoning	Spatial interpretation, problem-solving ability, ability to make decision, visual calculation	Frontal lobe
Response time	Ability to react to a series of movements	Ability to perform, vision-movement coordination, response capability	Whole area in prefrontal lobe, specific area in prefrontal lobe
Memory	Ability to recollect trifles in daily life	Long term memory, location memory, short term memory, learning ability	Area of hippocampus, area of central diencephalon

**Table 4:** Flow-chart of the six drills in the cognitive game.

Step	Training	Target abilities	How to play
1	Physical training	Hand tremor	To click/touch the numbers in order
2	Cognitive training	Memory	To memorize a card
3		Concentration	To find the same picture
4		Vision training	To find a new object
5		Recognition of icon images	To identify the meaning of an icon
6		Graphic interpretation	To interpret the graphs and explain the key points on the graph

**Table 5:** 6 cognitive exercise and each user interface.

Domain	Drills	User interaction	User Interface
Attention	Concentration training	To find the same picture	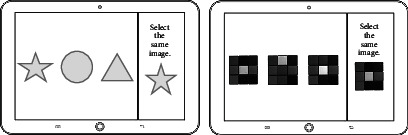
Logic	Identification training	To identify the meaning of an icon	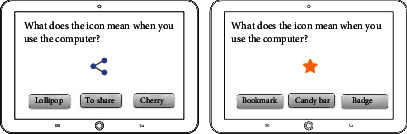
	Interpretation training	To interpret the graph	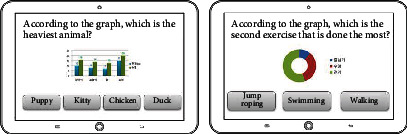
Response time	Vision adaptation training	To find a new object	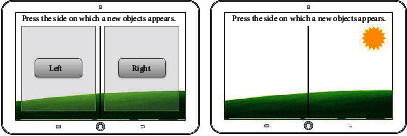
	Touch training	To touch the numbers in order	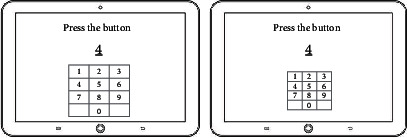
Memory	Memory training	To memorize the card	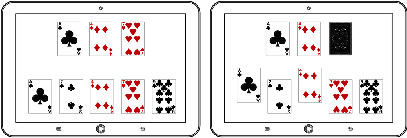

**Table 6:** Web-based game development environment.

Category	Program
Integrated development environment	Atom
Running environment	HTML5 browser
Equipment	PC and mobile device
Programming language	Typescript
BAAS	Firebase
Library	AngularJS, bootstrap, HammerJs, typescript

**Table 7:** Adjustment elements according to user's performance.

Mini games/drills	Screen	Adjustment elements
To find the same picture	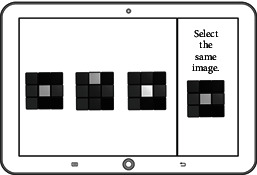	Sample number, sample similarity
To identify the meaning of icon	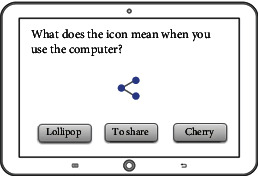	Level of abstraction in icon, frequency of icon usage
To interpret the graph	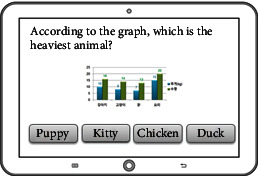	Complexity of graph, the difficulty of question
To find a new object	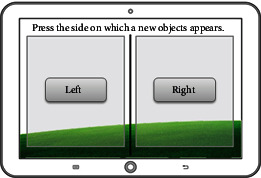	The size of a new object
To touch the numbers in order	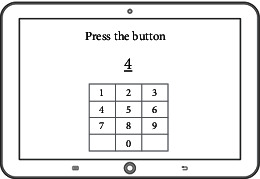	The size of number button
To memorize the card	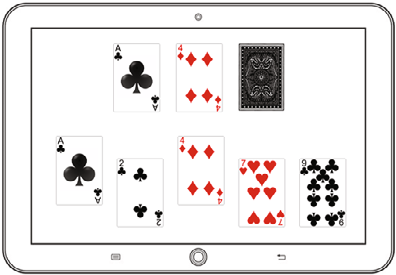	Number of card-in-question

**Table 8:** Survey information.

Subject	Older people in their 60s and 80s
Sample size	37 persons
Survey method	Face to face survey
Statistics program	SPSS, editing-coding-key‧ in-programming
Survey period	(i) pre-investigation: 2 days (February 15th and 19th, 2019) (ii) post-investigation: 1 day (April 19th, 2019)
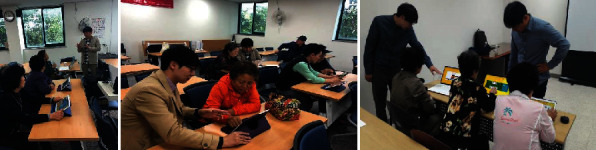	

**Table 9:** Survey participant's demographic information.

		Persons	Percent (%)
Total		(37)	100.0
Gender	Male	(5)	12.8
	Female	(32)	87.2

Age	Under 70s	(7)	17.9
	70-74	(14)	35.9
	75-79	(12)	30.8
	Over 80	(4)	15.4

**Table 10:** Survey results for the quality of life (before and after the cognitive exercise gameplay).

Item	(*n* = 37, by average point)		
	Pre	Post	Difference
Degree of pain and discomfort	2.14	2.32	0.19
Strength or energy	2.32	2.46	0.14
Sleep quality	2.49	2.62	0.14
Positive feeling (happiness, hope etc.)	2.59	2.78	0.19
Memory and concentration	2.27	2.49	0.22
Self-pride	2.57	2.86	0.30
Appearance	2.30	2.46	0.16
Negative feeling (depression, anxiety, despair etc.)	2.49	2.89	0.41
Ability to move	2.49	2.81	0.32
Ordinary activity	2.62	2.92	0.30
Ability to work	2.41	2.62	0.22
Family relationship and friendship	2.86	3.05	0.19
Social support and help	2.51	2.78	0.27
Sex life (*n* = 16)	1.94	2.31	0.37
Physical safety	2.51	2.51	0.00
The current living	2.89	2.78	-0.11
Available money or financial condition	2.70	2.62	-0.08
Public facilities (medical center and social welfare center)	2.84	3.11	0.27
New information or useful news for better living	2.70	2.84	0.14
Leisure activities and hobby	2.95	3.24	0.30
Living environment (climate, pollution, noise etc.)	2.19	2.27	0.08
Means of transportation	2.78	2.89	0.11
Personal faith or religious life	2.89	3.08	0.19
Overall health conditions	2.19	2.41	0.22
Overall quality of life	2.62	2.86	0.24

∗*p* < 0.1, ∗∗*p* < 0.05, and ∗∗∗*p* < 0.01.

**Table 11:** Survey results of geriatric depression (before and after cognitive exercise gameplay).

Question	(by %)			
	Pre (*n* = 37)		Post (*n* = 37)	
	Yes	No	Yes	No
Do you think that your current activities or enthusiasm decline significantly?	35.9	64.1	32.4	67.6
Do you feel your life is meaningless?	10.3	89.7	10.8	89.2
Do you often feel that your life is boring?	25.6	74.4	8.1	91.9
Do you usually feel pleasant?	15.4	84.6	13.5	86.5
Are you anxious about upcoming accidents?	17.9	82.1	13.5	86.5
Do you usually feel happy?	20.5	79.5	16.2	83.8
Do you often feel you have no hope?	10.3	89.7	13.5	86.5
Do you want to stay at home and feel reluctant to go out?	5.1	94.9	10.8	89.2
Do you feel your memory is inferior to that of your peers?	25.6	74.4	18.9	81.1
Do you think being alive is a joy?	7.7	92.3	5.4	94.6
Do you feel you are a worthless person?	5.1	94.9	8.1	91.9
Is your vigor good?	33.3	66.7	32.4	67.6
Do you feel your living has no hope?	23.1	76.9	21.6	78.4
Do you think your living status is worse than that of other people?	5.1	94.9	2.7	97.3

**Table 12:** Results of Korean version of MMSE for dementia screening (before and after cognitive exercise gameplay).

	(*n* = 37, by average point)		
	Pre-investigation	Post-investigation	Variation
Total score	4.81	5.05	0.24

∗*p* < 0.1, ∗∗*p* < 0.05, and ∗∗∗*p* < 0.01.

**Table 13:** Results of Korean version of MMSE for dementia screening for each question (before and after cognitive exercise gameplay).

Question	(by %)					
	Pre (*n* = 37)			Post (*n* = 37)		
	No	Sometimes (a little bit)	Frequently (a lot)	No	Sometimes (a little bit)	Frequently (a lot)
I do not know what month is today and what day of the week today	84.6	12.8	2.6	68.3	29.7	2.0
I cannot find the things I left	46.2	53.8	0.0	70.3	24.3	5.4
I make a promise and often forget it	79.5	20.5	0.0	70.3	29.7	0.0
I often go for something but forget to bring it	25.6	74.4	0.0	78.4	18.9	2.7
I have a difficulty in telling the names of things or men	38.5	59.0	2.6	24.3	73.0	2.7
I repeat to ask because I fail to understand in the middle of the conversation	46.2	51.3	2.6	29.7	67.6	2.7
I have experience of losing my way or moving around	84.6	15.4	0.0	62.2	37.8	0.0
My calculation abilities have declined	48.7	46.2	5.1	91.9	8.1	0.0
My personality has changed	59.0	35.9	5.1	37.8	51.4	10.8
I become poor at arranging things at home	89.7	10.3	0.0	94.6	5.4	0.0
I am unable to select and wear clothes on my own suited for a situation	94.9	5.1	0.0	83.8	16.2	0.0
I have a difficulty in arriving at a destination by public transportation on my own	94.9	5.1	0.0	100.0	0.0	0.0
Even when my underwear or clothes get dirty, I am unwilling to change them	94.9	2.6	2.6	100.0	0.0	0.0

**Table 14:** Satisfaction survey.

Question	(by points)
	Yes (*n* = 37)
Is training interesting?	4.54
Is instruction about how to train easy to understand?	4.59
Is training time adequate?	4.59
Do you think training difficulty level changes according to your performance?	4.32
Do you think icon training and graph training are helpful?	4.49
Do you think cognition training is helpful to advance your cognitive function?	4.65
Are you satisfied with the overall training?	4.57
Are you willing to recommend the training?	4.62
How difficult is the overall training?	3.19

**Table 15:** Improvement with regards to the game-based cognition training.

Improvement	Case (person)	Rate (%)
I want to be informed about how to use a computer	1	2.7
I want more immediately recognizable icon images	1	2.7
The same image is repeated	1	2.7
I want to have more training time	1	2.7
Training questions are too simple	1	2.7
I need a more detailed description of difficult training	1	2.7
Changes are needed	1	2.7
There is nothing particular	29	81.1

**Table 16:** Performance results of the proposed cognitive game by each training.

Play time	(*n* = 37, by point)												
	Number of people	Touch training		Concentration training		Memory training		Vision adaptation training		Icon training		Graph training	
		Average	Standard deviation	Average	Standard deviation	Average	Standard deviation	Average	Standard deviation	Average	Standard deviation	Average	Standard deviation
1	32	6.00	0.00	5.31	0.95	4.72	1.18	5.53	0.71	3.13	1.56	4.25	1.06
2	37	5.95	0.22	4.89	0.97	4.58	1.21	5.08	0.98	2.61	1.33	5.13	1.08
3	37	5.78	0.74	5.03	0.97	4.70	1.21	5.46	0.79	3.65	1.49	5.08	1.17
4	36	5.89	0.39	4.83	1.09	4.61	1.25	5.36	0.92	3.86	1.32	5.31	0.94
5	31	5.90	0.39	5.00	1.02	4.45	1.27	5.71	0.45	3.35	1.38	5.35	0.82
6	35	5.97	0.17	5.06	0.92	4.89	1.14	5.60	0.68	3.94	1.66	5.31	0.82
7	32	5.94	0.24	5.03	1.05	4.41	1.22	5.66	0.54	4.19	1.21	5.44	0.83
8	34	5.94	0.24	5.21	0.76	4.82	1.07	5.82	0.38	3.97	1.36	5.41	0.84
9	32	5.94	0.24	5.03	1.05	4.69	1.04	5.66	0.59	4.06	1.22	5.22	1.36
10	34	6.00	0.00	5.29	0.75	4.71	1.20	5.91	0.28	4.35	1.13	5.41	1.09
11	36	5.97	0.16	5.03	1.01	4.64	1.06	5.64	0.58	4.61	1.03	5.47	0.87
12	34	5.91	0.28	5.15	0.84	4.94	1.00	5.79	0.53	4.47	1.24	5.35	0.90
13	37	6.00	0.00	5.11	0.92	5.16	0.89	5.86	0.34	4.76	1.00	5.65	0.58
14	35	5.91	0.28	5.23	0.83	4.86	1.07	5.80	0.40	4.91	0.87	5.54	0.73
15	33	6.00	0.00	5.27	0.79	5.09	0.83	5.79	0.41	4.61	1.20	5.45	0.74
16	32	5.91	0.29	5.09	0.88	5.00	0.90	5.75	0.66	4.34	0.89	5.38	1.22
Total	**37**	**5.94**	**0.30**	**5.09**	**0.94**	**4.77**	**1.12**	**5.65**	**0.65**	**4.05**	**1.40**	**5.30**	**1.00**

**Table 17:** Memory training-fixed effect estimation.

Parameter	Estimation	Standard error	*t*	Significance probability	95% confidence interval	
					Lowest limit	Upper limit
Intercept	4.532	0.126	36.053	.000	4.282	4.781
Times	0.026	0.009	2.764	.006	0.007	0.044

**Table 18:** Vision adaptation training-fixed effect estimation.

Parameter	Estimation	Standard error	*t*	Significance probability	95% confidence interval	
					Lowest limit	Upper limit
Intercept	5.353	0.060	89.171	.000	5.234	5.471
Times	0.034	0.006	5.941	.000	0.023	0.045

**Table 19:** Icon training-fixed effect estimation.

Parameter	Estimation	Standard error	*t*	Significance probability	95% confidence interval	
					Lowest limit	Upper limit
Intercept	3.081	0.140	22.003	.000	2.804	3.359
Times	0.113	0.011	10.419	.000	0.092	0.134

**Table 20:** Graph training-fixed effect estimation.

Parameter	Estimation	Standard error	*t*	Significance probability	95% confidence interval	
					Lowest limit	Upper limit
Intercept	4.938	0.115	43.016	.000	4.709	5.166
Times	0.042	0.008	5.423	.000	0.027	0.057

## Data Availability

The data presented in this study are available on request from the corresponding author.
